# The influence of empathy with nature on pro-environmental behaviors in young children: evidence from behavioral and eye-tracking studies

**DOI:** 10.3389/fpsyg.2026.1778192

**Published:** 2026-03-12

**Authors:** Zhengyu Yuan, Guohua Zhou

**Affiliations:** 1 School of Education Science, Hunan Normal University, Changsha, China; 2 Cognition and Human Behavior Key Laboratory of Hunan Province, Changsha, China

**Keywords:** attentional bias, empathy with nature, eye-tracking, pro-environmental behavior, young children

## Abstract

**Introduction:**

The early cultivation of pro-environmental behavior is crucial for sustainable development, yet the mechanisms driving such behavior in early childhood—especially affective factors like empathy with nature—remain underexplored.

**Methods:**

Through two experiments combining behavioral and eye-tracking measures, this study examined the effect of empathy with nature on pro-environmental behavior in 4–6-year-olds. Experiment 1 employed a 2 (empathy induced vs. control) × 3 (age: 4, 5, 6 years) between-subjects design with 180 children, measuring donations of stickers and candies to an environmental organization. Experiment 2 recruited 61 five-year-olds and used eye-tracking to investigate the attentional mechanism behind the empathy-behavior link.

**Results:**

Experiment 1 revealed a significant main effect of empathy induction, with children in the empathy condition donating more than controls, and a significant main effect of age, indicating increased donations with age; the interaction was non-significant, suggesting stable empathy effects across ages. Experiment 2 replicated the behavioral effect and showed that children in the empathy condition had significantly higher ratios of total fixation duration and count on the pro-environmental donation option. Both fixation ratios were strongly positively correlated with donation amounts (*r*s > 0 .79).

**Discussion:**

These findings demonstrate that empathy with nature is a key affective promoter of pro-environmental behavior in young children, and its mechanism is partially mediated by enhanced visual attentional bias toward pro-environmental options. The study provides new process-level evidence for the empathy–environmentalism link in early development and offers empirical support for empathy-based environmental education practices.

## 1 Introduction

Pro-environmental behavior in young children, a crucial extension of prosocial behavior into the ecological domain, holds fundamental significance for cultivating early awareness of sustainable development and fostering intergenerational environmental action (Kellert, 2002; Stern, 2000). Regarding assessment tools, previous research on adult pro-environmental behavior primarily employs self-report questionnaires (e.g., ecological behavior scales; Kaiser, 1998) or field observation methods (e.g., recycling behavior monitoring; Steg and Vlek, 2009). For young children, studies often adapt paradigms from prosocial behavior research, using donation tasks, sharing games, or behavioral choice experiments to measure their pro-environmental tendencies (Eisenberg et al., 2006; Damon et al., 2006; Erdogan et al., 2012). Specifically, in the 4–6-year-old stage, pro-environmental behavior shows distinct developmental characteristics: 4-year-olds begin to exhibit initial altruism and environmental concern, though their behaviors are often influenced by immediate context; by ages 5–6, with the rapid development of theory of mind, empathy, and executive functions, children’s pro-environmental behaviors (e.g., resource sharing, waste cleanup) become more stable and proactive (Blake et al., 2014; Knafo et al., 2008; Malti et al., 2012). However, existing research has predominantly focused on adults or school-aged children. Investigations into the mechanisms during the critical 4–6-year-old period, when pro-environmental behaviors begin to emerge, remain insufficient, particularly lacking empirical evidence integrating cognitive and affective processes. Therefore, this study aims to examine the influence of empathy with nature on pro-environmental behavior in 4–6-year-olds, addressing this empirical gap and providing a theoretical basis for early environmental education.

Empathy with nature, defined as an individual’s emotional resonance and cognitive understanding of the natural environment, is a key affective antecedent predicting pro-environmental behavior (Schultz, 2000; Tam, 2013). Empathy with nature was selected as the core variable to investigate children’s pro-environmental behavior for two main reasons. First, empathy is a fundamental motivational basis for prosocial behavior (Eisenberg et al., 2006), and pro-environmental behavior is considered an extension of prosocial behavior into the ecological domain (Kals et al., 1999). Second, compared to theoretical models in adult research that emphasize moral norms or cost-benefit calculations (e.g., norm activation theory; Stern, 2000), young children’s pro-environmental behavior is more likely to be driven by direct emotional experiences. Empathy with nature provides a psychological mechanism that connects external environmental states with internal affective responses (Cheng and Monroe, 2012)

Empathy with nature is typically conceptualized as a multidimensional construct encompassing affective care for nature (e.g., sympathy for damaged nature), perspective-taking (understanding nature’s plight), and empathetic concern (Berenguer, 2007). In terms of measurement, adult studies commonly use self-report scales (e.g., adapted versions of the Empathic Concern Scale; Schultz, 2001), whereas research with young children relies more on behavioral experimental paradigms. These often employ scenario stories, pictures, or videos to induce a state of empathy with nature, followed by observation or measurement of subsequent behavior (Collado et al., 2013). The “induced vs. non-induced empathy with nature” manipulation used in this study belongs to this experimental priming paradigm, aiming to elicit children’s empathetic response by presenting contrasting scenarios of nature being harmed (e.g., deforestation) versus unharmed.

Research indicates that empathy with nature can effectively promote environmental intentions and behaviors (e.g., resource conservation, participation in clean-up activities) in both adults and children (Sevillano et al., 2007). However, studies specifically targeting the 4–6-year-old age group are scarce, despite this period being a critical stage for the rapid development of empathy and the internalization of social norms (Decety and Svetlova, 2012). Investigating the impact of empathy with nature on young children’s pro-environmental behavior cannot only reveal the affective driving mechanisms behind early environmental actions—addressing the current research focus on cognition over emotion—but also provide empirical evidence for designing emotion-arousing environmental education programs for young children.

Although behavioral measurement is a direct way to assess children’s pro-environmental behavior, it falls short of revealing the underlying immediate cognitive and affective processes. Eye-tracking technology offers an effective solution, as its high temporal resolution and minimal invasiveness make it an ideal tool for exploring the mechanisms of social cognition and affective response in young children (Franchak et al., 2011; Hessels, 2020). In the context of empathy with nature, eye-tracking metrics (e.g., fixation duration, fixation count) can sensitively reflect an individual’s attention allocation and emotional engagement with specific visual stimuli (e.g., images of damaged natural elements or logos of environmental organizations in need). These processes are considered precursors to empathetic response and subsequent decision-making (e.g., donating) (Bollen et al., 2025). Research shows that gaze patterns toward affective or morally relevant stimuli are associated with an individual’s level of empathy and prosocial tendencies (Hessels et al., 2020), providing a theoretical basis for using eye-tracking to explore how empathy with nature guides children’s attentional resources and thereby influences their pro-environmental behavior.

Therefore, integrating behavioral and eye-tracking methods to jointly examine the influence of empathy with nature on children’s pro-environmental behavior offers significant methodological advantages. Behavioral data (e.g., donation quantity) provide conclusive evidence regarding whether and to what extent pro-environmental behavior occurs (Eisenberg et al., 2006). In contrast, eye-tracking data can uncover the how—the potential mechanism behind this behavioral outcome. That is, they can reveal whether and how inducing empathy with nature alters children’s visual attention patterns toward environment-related information (e.g., increasing gazes toward the environmental organization), thereby linking external affective priming with internal cognitive processing (Decety, 2021; Eslinger et al., 2002). This multi-method convergent strategy not only enhances the validity of the research conclusions but also transcends the limitations of traditional behavioral measures. It allows for a deeper elucidation of the cognitive pathway through which empathy with nature affects children’s pro-environmental behavior, providing empirical support for constructing more comprehensive theoretical models.

In summary, the early cultivation of pro-environmental behavior is crucial, yet the mechanism of action of empathy with nature—a key affective driver—remains insufficiently understood in early childhood. Existing research has the following shortcomings: First, most studies focus on adults or adolescents, leaving a relative scarcity of empirical research targeting the critical 4–6-year-old period when pro-environmental attitudes and behaviors begin to emerge (Collado et al., 2013). Second, existing research predominantly uses questionnaires or single behavioral measures, making it difficult to reveal the immediate cognitive processing through which empathy with nature influences pro-environmental behavior. Finally, there is a lack of multi-method evidence integrating behavioral performance with underlying attentional mechanisms to comprehensively delineate the complete pathway from affective priming to behavioral output.

To address these gaps, this study aims to systematically investigate the influence of empathy with nature on pro-environmental behavior in 4–6-year-olds and its underlying attentional mechanism through two experiments. Experiment 1 serves as a foundational behavioral investigation, aiming to establish causal evidence for whether inducing empathy with nature can effectively promote pro-environmental behavior (measured by donation quantity) across different age groups (4-, 5-, and 6-year-olds), and to examine developmental differences in this effect. Building upon and extending the behavioral findings, Experiment 2 employs eye-tracking technology to delve into the cognitive mechanism—specifically, whether empathy with nature influences pro-environmental behavior by enhancing children’s visual attentional bias toward pro-environmental options. This two-experiment design follows a logical progression from behavioral demonstration (Experiment 1) to process-level mechanistic explanation (Experiment 2), with each study making an independent and essential contribution to the overall research aim.

## 2 Experiment 1: the influence of empathy with nature on pro-environmental behavior in young children

### 2.1 Purpose and hypotheses

Experiment 1 aimed to examine the combined influence of induced empathy with nature and age on pro-environmental behavior (measured by donation quantity) in young children, using a picture-based priming paradigm to manipulate their state of empathy with nature. A 2 (Condition: empathy with nature induced vs. not induced) × 3 (Age: 4- vs. 5- vs. 6-year-olds) between-subjects factorial design was employed.

Based on empathy-prosocial behavior theory and related research (Eisenberg et al., 2006; Schultz, 2000), as well as the developmental trend of increasing altruistic behavior with age in early childhood (Blake et al., 2014), the following hypotheses were proposed:

*H1*: Children in the induced-empathy condition would donate significantly more stickers than those in the control condition.

*H2*:Children in the induced-empathy condition would donate significantly more candies than those in the control condition.

*H3*: Donation behavior would differ significantly across age groups, showing a pattern of 6-year-olds > 5-year-olds > 4-year-olds (for both stickers and candies).

*H4*: Donation behavior would differ significantly across age groups, showing a pattern of 6-year-olds > 5-year-olds > 4-year-olds (for both stickers and candies).

### 2.2 Pilot study: validation of the empathy priming materials

A pilot study was conducted to validate the effectiveness of the empathy-with-nature priming materials used in both Experiments 1 and 2. The development of the priming materials was informed by Kim’s method for manipulating empathy with nature (i.e., using images of injured animals) and drew upon visual stimuli from related research (e.g., Wang et al., 2023; Kim and Seock, 2019). The materials for the induced-empathy condition consisted of four pictures depicting animals suffering due to environmental issues (e.g., a seal entangled in a fishing net, birds contaminated by oil). The control condition materials consisted of four corresponding pictures showing the same animals in their normal, undisturbed state.

Seventy-two children (55.56% boys, 44.44% girls) from a kindergarten in Changsha City were recruited for the pilot study. They were randomly assigned to either Group A (induced empathy) or Group B (control). Each group viewed their respective set of pictures. After viewing each picture, children immediately completed a simplified state empathy scale. This scale, adapted from Berenguer (2010) and Sheng et al. (2021), included four items (e.g., “After seeing the picture, I can feel what the animal in the picture feels”). Responses were collected using a child-friendly 3-point pictorial scale (thumbs down = 1 point, one thumb up = 2 points, two thumbs up = 3 points), yielding a total score ranging from 4 to 12 (1–3 points per item across 4 items).

An independent-samples *t*-test was conducted on the total empathy scores of the two groups. The results showed that the score for Group A (induced empathy, *M* = 10.61, *SD* = 1.15) was significantly higher than that for Group B [control, *M* = 6.72, *SD* = 1.15), *t*(70) = 14.526, *p* < .001, 95% CI (3.37, 4.47)]. This indicates that the selected picture materials were effective in inducing the intended state of empathy with nature in the target age group, validating the priming manipulation.

### 2.3 Materials main experiment

#### 2.3.1 Participants

A priori power analysis using G*Power 3.1 (Faul et al., 2007) indicated that a minimum of 179 participants was required to detect a medium effect (*f* = 0.25) with 80% power (α = 0.05) in a 2 × 3 between-subjects ANOVA. We recruited 180 healthy children aged 4–6 years from a kindergarten in Changsha, China. All participants were right-handed, had normal or corrected-to-normal vision, and had no history of neurological or psychiatric disorders. Due to parental concerns about dental caries, one 4-year-old did not complete the candy donation task, resulting in a valid sample of 179 for that measure. The final sample comprised 60 4-year-olds (M = 53.2 months, SD = 3.4; 31 boys, 29 girls), 60 5-year-olds (M = 65.1 months, SD = 3.6; 31 boys, 29 girls), and 60 6-year-olds (M = 76.8 months, SD = 3.9; 29 boys, 31 girls). Within each age group, children were randomly assigned to either the control or induced-empathy condition. A chi-square test confirmed no significant gender differences across conditions, χ^2^(5) = 0.56, *p* = 0.46. The study protocol was approved by the University’s Institutional Review Board (Approval No.: 526). Written informed consent was obtained from guardians, and verbal assent was obtained from each child.

#### 2.3.2 Experimental design

A 2 (Condition: induced empathy vs. control) × 3 (Age: 4, 5, and 6 years) between-subjects factorial design was employed. The independent variables were empathy priming condition and age group. Condition was manipulated by presenting children with either empathetically evocative images of animals suffering from environmental degradation or neutral images of animals in their natural habitats. Age was included as a factor to examine developmental trends in pro-environmental behavior and to test whether the effect of empathy induction varied across the preschool years. The dependent variable was children’s pro-environmental behavior, operationalized as the number of stickers and candies (each ranging from 0 to 5) that children voluntarily placed into a brown envelope designated for donation to an environmental organization. Both stickers and candies were used as donation items to assess whether the observed effects generalized across different types of personally valued resources, thereby enhancing the robustness of the behavioral measure.

#### 2.3.3 Experimental materials

##### 2.3.3.1 Empathy priming materials

The priming stimuli were identical to those validated in the pilot study and consisted of eight full-color animal pictures. For the induced-empathy condition, four images depicted animals suffering as a consequence of human-induced environmental degradation, including a seal entangled in discarded fishing nets, birds contaminated by an oil spill, a polar bear stranded on melting ice, and a sea turtle ensnared in a plastic bag. These images were selected to elicit empathetic concern by vividly illustrating harm to natural entities. For the control condition, four corresponding images portrayed the same animal species in their natural, undisturbed habitats (e.g., a seal resting on an ice floe, birds perching on a clean shoreline), thereby providing a neutral visual baseline that controlled for the presence of animals and environmental themes without evoking empathetic distress (Sheng et al., 2021).

##### 2.3.3.2 Pro-environmental behavior measurement materials

To assess pro-environmental behavior, we employed a resource donation paradigm adapted from developmental studies of prosocial behavior. Donation items consisted of two types of personally valuable resources: stickers and candies. Stickers (Haibite brand, cartoon style, appropriate for ages 3–6 years) were selected for their high attractiveness to young children; multiple sheets were prepared, each containing seven distinct cartoon patterns. Candies (Alpenliebe brand, individually wrapped, available in milk, strawberry, and grape flavors) were chosen as a commonly desired edible reward. Two types of envelopes were used to operationalize the donation choice: a white envelope (10 cm × 7 cm) designated for items the child would “take home,” and a brown envelope of identical size designated for items to “donate to the environmental organization to help the animals.” Additionally, a set of “Help the Animal Home” path-finding cards was used as a warm-up activity to build rapport with each child and to introduce the environmental protection theme in a developmentally appropriate manner before the formal experimental procedure began.

#### 2.3.4 Procedure

All experimental sessions were conducted individually in a quiet, well-lit room within the kindergarten by a trained female experimenter experienced in working with young children. Each session lasted approximately 8–10 min and followed a standardized four-phase procedure designed to be developmentally appropriate and engaging while maintaining experimental control.

##### 2.3.4.1 Warm-up and introduction phase

To establish rapport and introduce the environmental theme naturally, the experimenter began by playing a simple “Help the Animal Home” path-finding game with the child. During this interaction, the experimenter identified herself as a staff member from an environmental organization dedicated to helping animals. Upon successful completion of the game, the child was rewarded with five stickers and five candies, with the experimenter explicitly stating, “These are your rewards for completing the task; they all belong to you.” This initial endowment established the child’s ownership over the resources that would later be available for donation.

##### 2.3.4.2 Empathy priming phase

Following random assignment, children proceeded to either the induced-empathy or control condition. In the induced-empathy condition, the experimenter presented the four suffering animal images sequentially, accompanied by a standardized verbal prompt designed to facilitate empathetic engagement: “Look at this picture. What’s happening to the little animal? Do you think it feels happy or unhappy? Can you understand how it might be feeling?” After viewing each image, children briefly indicated their emotional response using the same 3-point pictorial scale (thumbs down, one thumb up, two thumbs up) employed in the pilot study. This brief reporting served both to verify comprehension and to reinforce the empathy induction. In the control condition, children viewed the four neutral animal images with a parallel prompt focused on objective description rather than emotional resonance: “Look at this picture. What is the little animal doing? How do you feel about it?” They then provided the same brief feeling report to maintain procedural consistency across conditions.

##### 2.3.4.3 Donation task phase

Pro-environmental behavior was measured using an adapted version of the Dictator Game paradigm, modified for use with young children. The experimenter introduced two envelopes—a white envelope labeled “for me to take home” and a brown envelope labeled “for me to donate to the environmental organization to help the animals.” The experimenter emphasized that the child was free to decide whether to donate and how many items to donate, and assured the child that their decision would remain private (“This is our secret; your teacher and parents will not know”). This privacy assurance was designed to minimize social desirability effects. The donation task consisted of two subtasks presented in counterbalanced order across participants. In the sticker donation task, the experimenter placed the child’s five pre-selected stickers on the table, then excused herself and left the room, observing unobtrusively from outside to allow the child to allocate the stickers independently. After the child finished placing stickers into the two envelopes, the experimenter returned. The candy donation task followed an identical procedure using the five pre-selected candies.

##### 2.3.4.4 Conclusion phase

Upon completion of both donation tasks, the experimenter collected the brown envelope containing the donations. The white envelope, containing the items the child had chosen to keep, was given to the child. The experimenter thanked and praised the child for their participation before escorting them back to their classroom.

**FIGURE 1:**
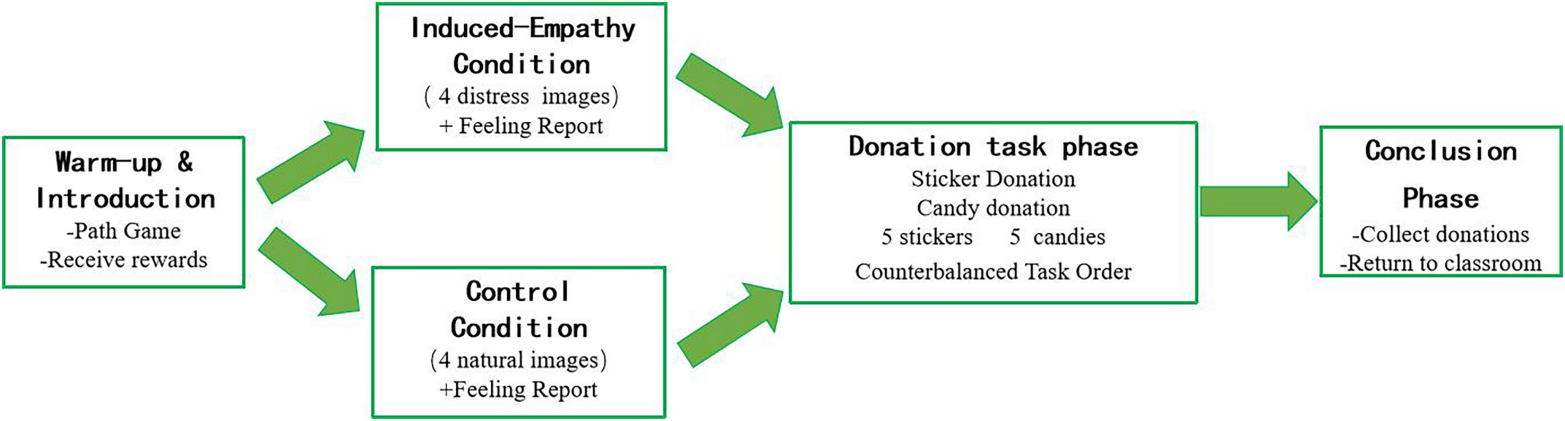
Flowchart of the experimental procedure for Experiment 1.

Figure 1 presents a flowchart summarizing the key stages of the experimental procedure for Experiment 1, including the warm-up phase, empathy priming phase (with condition-specific pathways), donation task phase (with counterbalanced sticker and candy subtasks), and conclusion phase.

#### 2.3.5 Data analysis

All statistical analyses were conducted using SPSS Version 27.0 (IBM Corp., Armonk, NY). For the sticker (candy) donation data, a 2 (Condition: induced empathy vs. control) × 3 (Age: 4, 5, 6 years) between-subjects analysis of variance (ANOVA) was performed to examine the main effects of empathy induction and age, as well as their potential interaction, on children’s pro-environmental behavior. Following significant main effects, *post-hoc* comparisons were conducted using Tukey’s Honestly Significant Difference (HSD) test to identify specific group differences while controlling for Type I error. Additionally, a paired-samples *t*-test was conducted on the full sample to compare donation rates across the two resource types (stickers vs. candies), thereby testing the cross-material consistency of children’s pro-environmental behavior.

### 2.4 Results and analysis

#### 2.4.1 Donation of stickers under different conditions of empathy with nature and age

To examine the effects of empathy priming and age on children’s pro-environmental behavior, a two-way between-subjects analysis of variance (ANOVA) was first conducted with the number of stickers donated as the dependent variable. The factors were Condition (2 levels: induced empathy vs. control) and Age (3 levels: 4-, 5-, and –6-year-olds). Descriptive statistics are presented in Table 1.

**TABLE 1:** Means and standard deviations for stickers donated across age and condition.

Age	No natural empathy group		Natural empathy group	
	M	SD	M	SD
4-year-olds	1.00	0.85	1.84	1.37
5-year-olds	1.83	1.13	2.68	1.62
6-year-olds	2.03	1.24	3.10	1.69

**FIGURE 2:**
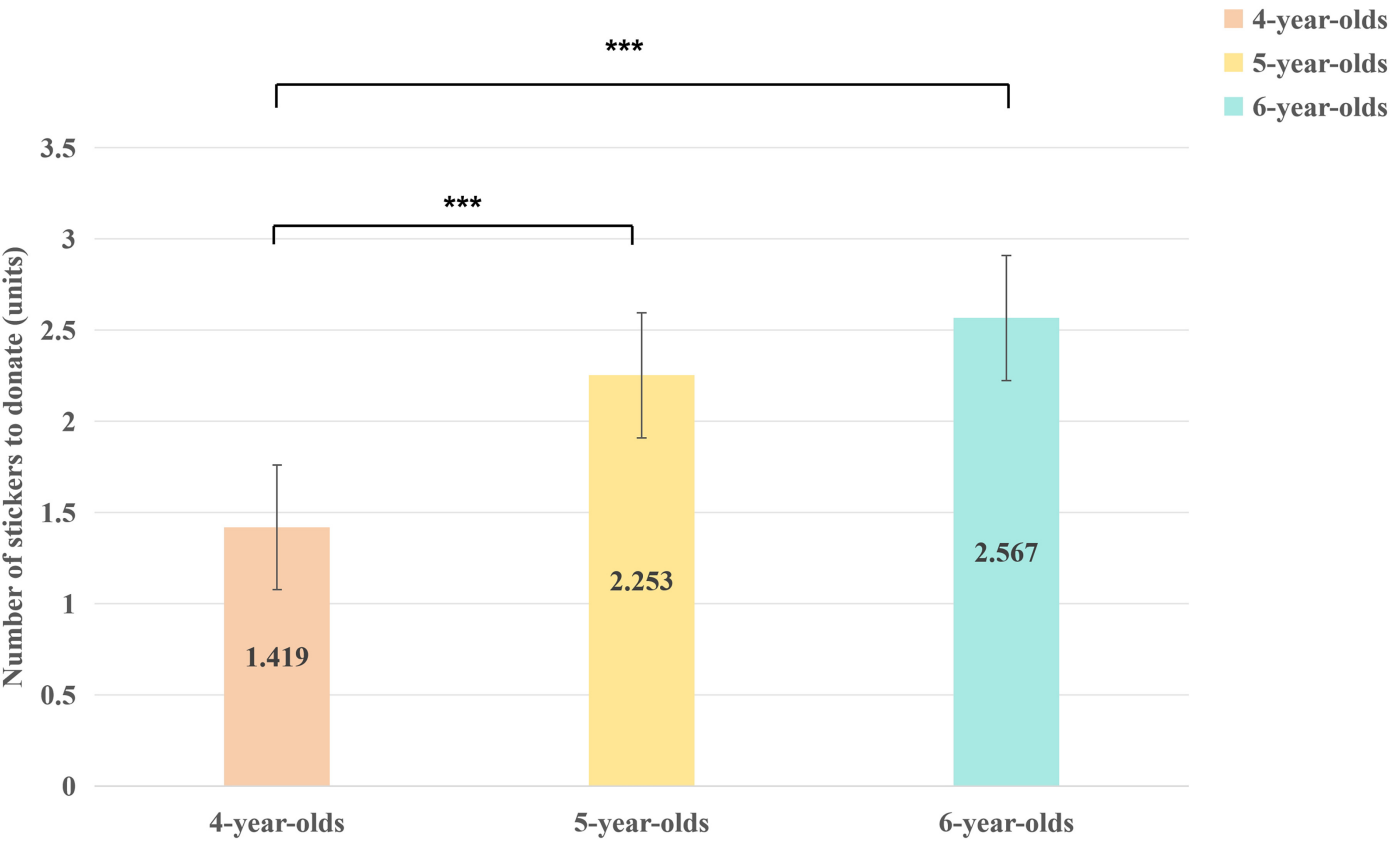
The difference of the number of stickers donated by children of different ages (***p < 0.001).

The ANOVA revealed a significant main effect of Age, *F*(2, 174) = 11.50, *p* < 0.001, $\eta_{p}^{2}$ = 0.12. The main effect of Condition was also significant, *F*(1, 174) = 20.70, *p* < 0.001, $\eta_{p}^{2}$ 0.11. The Age × Condition interaction was not significant, *F*(2, 174) = 0.14, *p* = 0.87, $\eta_{p}^{2}$ < 0.01. Given the non-significant interaction, follow-up analyses were conducted on the two main effects. For the significant main effect of Age, Tukey’s HSD *post hoc* comparisons showed that both 5-year-olds (*M* = 2.25, *SD* = 1.44) and 6-year-olds (*M* = 2.57, *SD* = 1.54) donated significantly more stickers than 4-year-olds (*M* = 1.42, *SD* = 1.22), all *p* < 0.001. The difference between 5- and 6-year-olds was not significant (*p* = 0.874) (see Figure 2).

**FIGURE 3:**
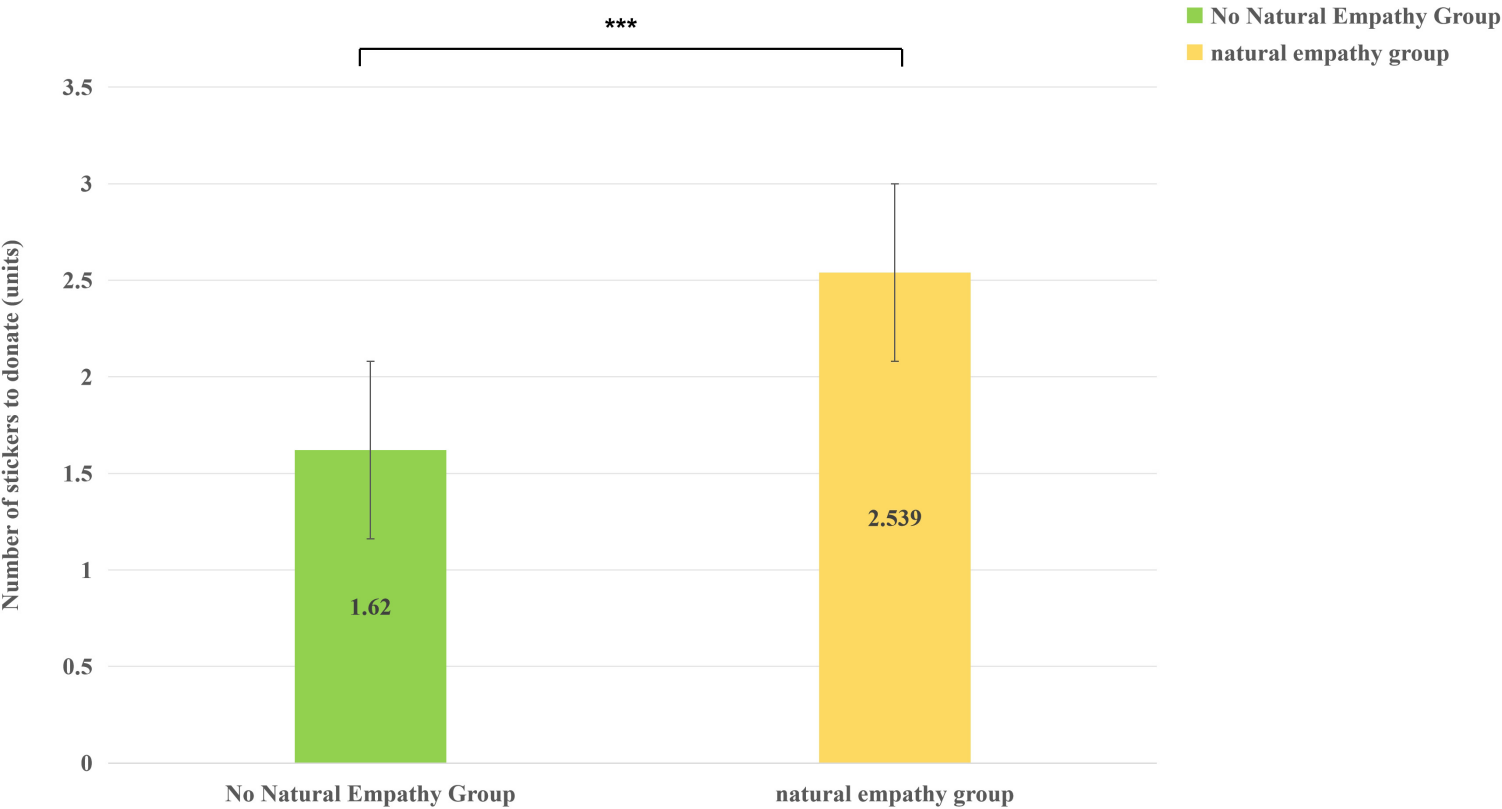
The difference of children’s donated stickers in different situations (***p < 0.001).

Analysis of the significant main effect of Condition indicated that children in the induced-empathy condition donated significantly more stickers (*M* = 2.54, *SD* = 1.64) than those in the control condition (*M* = 1.62, *SD* = 1.14) (see Figure 3).

#### 2.4.2 Donation of candies under different conditions of empathy with nature and age

An identical 2 × 3 ANOVA was conducted with the number of candies donated as the dependent variable. The valid sample size was 179 for this analysis, as one 4-year-old did not participate in the candy donation task. Descriptive statistics are presented in Table 2.

**TABLE 2:** Means and standard deviations for candies donated across age and condition.

Age	No natural empathy group		natural empathy group	
	M	SD	M	SD
4-year-olds	1.29	1.08	1.71	1.40
5-year-olds	1.69	1.69	2.71	1.19
6-year-olds	1.97	1.07	3.03	1.16

The ANOVA results showed a significant main effect of Age, *F*(2, 173) = 11.77, *p* < 0.001, $\eta_{p}^{2}$ = 0.12, and a significant main effect of Condition, *F*(1, 173) = 23.48, *p* < 0.001, $\eta_{p}^{2}$ = 0.12. The Age × Condition interaction was not significant, *F*(2, 173) = 1.43, *p* = 0.24, $\eta_{p}^{2}$ = 0.02.

**FIGURE 4:**
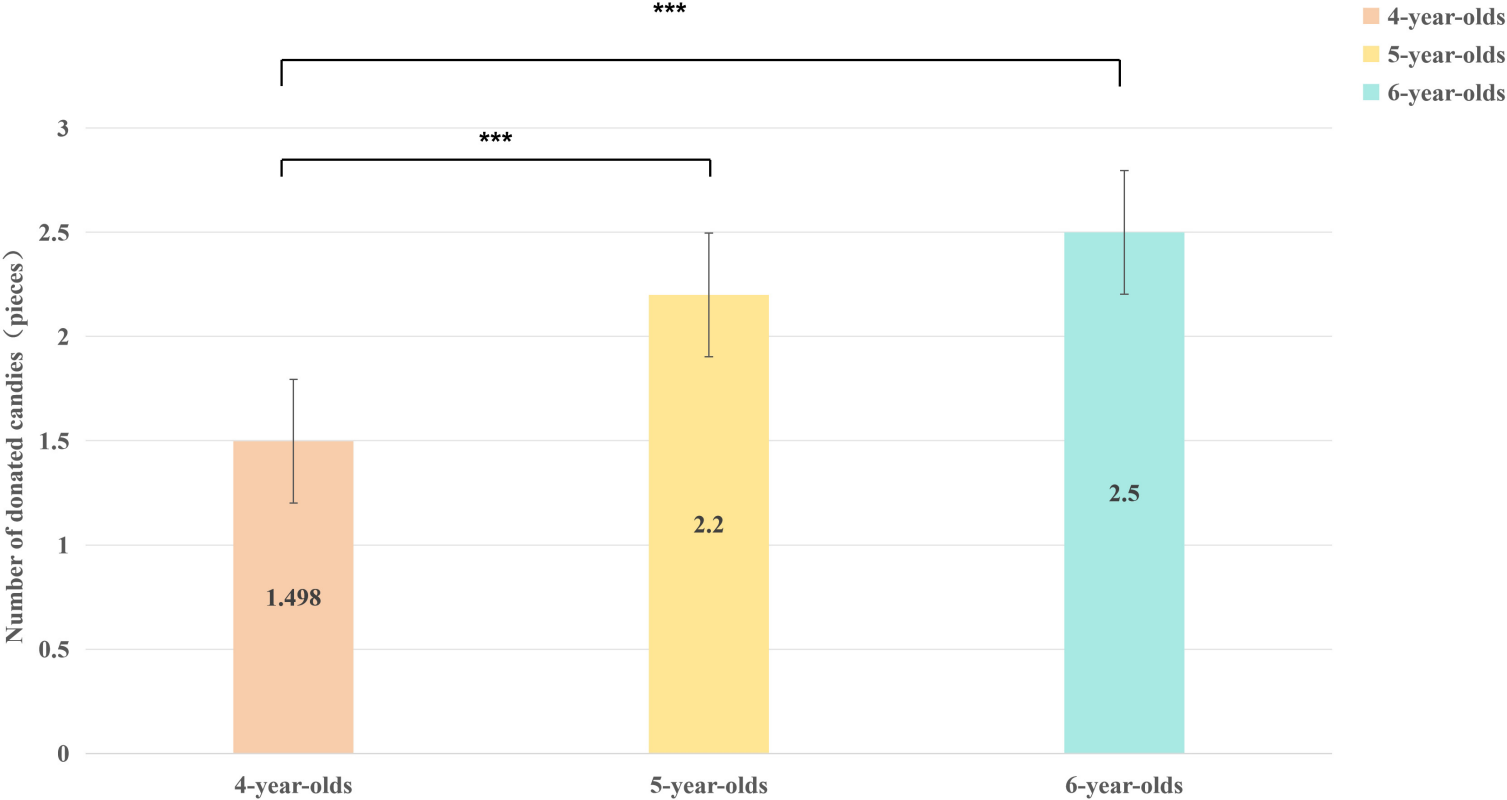
The difference of the number of Candies donated by children of different ages (***p < 0.001).

Follow-up analyses on the main effects were conducted. Tukey’s HSD post hoc tests for the Age main effect revealed that 5-year-olds (*M* = 2.20, *SD* = 1.32) and 6-year-olds (*M* = 2.50, *SD* = 1.30) donated significantly more candies than 4-year-olds (*M* = 1.50, *SD* = 1.26), *p* = 0.001 and *p* < 0.001, respectively. The difference between 5- and 6-year-olds was not significant *(p* = 0.16) (see Figure 4).

**FIGURE 5:**
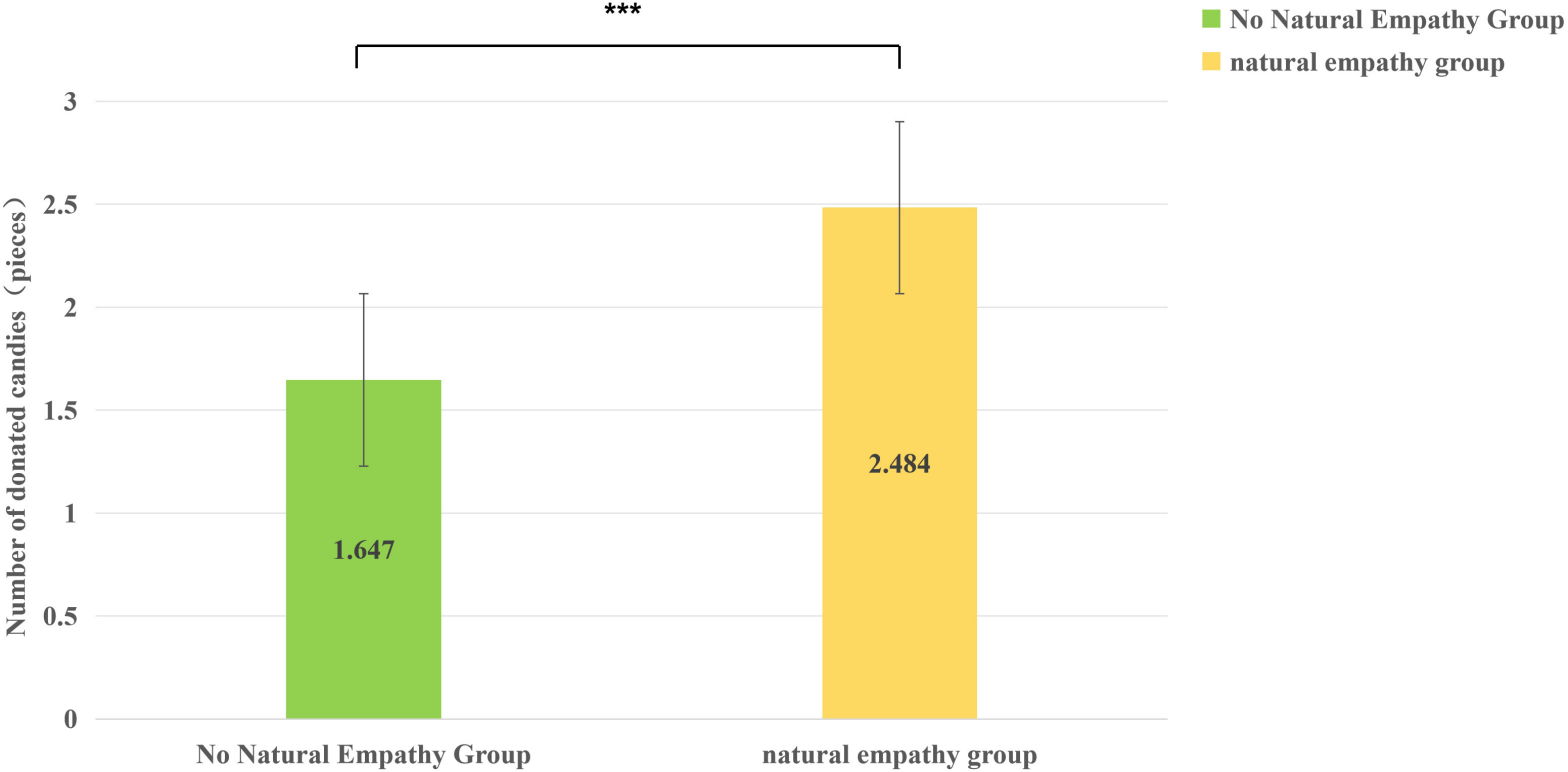
The difference of children’s donated candies in different situations (***p < 0.001).

For the main effect of Condition, children in the induced-empathy condition donated significantly more candies (*M* = 2.50, *SD* = 1.41) than those in the control condition (*M* = 1.65, *SD* = 1.30) (see Figure 5).

#### 2.4.3 Donation of candies under different conditions of empathy with nature and age

A paired-samples *t*-test was conducted on the whole sample to examine whether the type of donation item (stickers vs. candies) influenced children’s pro-environmental behavior. The results showed no significant difference between the number of stickers donated (*M* = 2.10, *SD* = 1.49) and the number of candies donated (*M* = 2.08, *SD* = 1.27), *t*(178) = 0.18, *p* = 0.86,*95% CI* (−0.17, 0.20). This indicates that children’s pro-environmental behavior, as measured in this paradigm, was consistent across different types of rewards, demonstrating cross-material stability.

### 2.5 Discussion

Experiment 1 systematically examined the effects of induced state empathy with nature and age on young children’s pro-environmental behavior by manipulating empathy and measuring resource donation. The results clearly supported the main hypotheses, providing direct causal evidence for understanding the early drivers of pro-environmental behavior in children and revealing important developmental characteristics.

First, the results strongly supported the core hypothesis that empathy with nature is a key factor driving children’s pro-environmental behavior (H1 and H2). Children in the induced-empathy condition donated significantly more of both stickers and candies than those in the control condition. This finding aligns with conclusions from adult studies (Schultz, 2000; Berenguer, 2007) and successfully extends the “empathy-altruism” hypothesis (Batson, 2014) to the domain of young children’s caring behavior toward non-human natural entities. It demonstrates that even in preschoolers whose cognitive abilities are still developing, emotional resonance (i.e., affective empathy) elicited by visual cues is sufficient to motivate decisions to sacrifice personally valuable resources for environmental protection. This confirms that emotional arousal may be more fundamental and effective than purely cognitive instruction in early childhood environmental education.

Second, a significant main effect of age was found. Overall, 5- and 6-year-olds demonstrated a significantly higher level of pro-environmental donation than 4-year-olds. This is consistent with our developmental hypothesis (H3) and the general trend of increasing prosocial behavior with age (Eisenberg et al., 2006; Lamb and Lerner, 2015). This growth likely stems from the concurrent development of multiple capacities: children’s empathic abilities, particularly the cognitive empathy (perspective-taking) component, continue to develop in the late preschool years (Decety and Svetlova, 2012), enabling a deeper understanding of the plight of environmental victims. Furthermore, improvements in executive functions (e.g., impulse control, delay of gratification) help children make more altruistic choices between immediate personal gain and future/other-oriented environmental benefits (Blake et al., 2014). However, it is noteworthy that the difference between 5- and 6-year-olds was not significant. This may suggest a period of relatively marked growth in pro-environmental behavior between ages 4 and 5, followed by a more stable plateau between 5 and 6. The specific cognitive mechanisms underlying this pattern warrant further investigation with more refined measures.

Third, the non-significant interaction between empathy and age is an important finding. It indicates that within the 4–6-year-old range examined, the promoting effect of empathy induction is consistent across age groups. In other words, even 4-year-old children’s pro-environmental behavior can be significantly enhanced by empathy priming. This highlights the efficacy and fundamental role of the affective-driven pathway in early life. This result also implies that empathy-arousal strategies hold potential value in environmental education for different age groups within this range, and the core intervention logic may have cross-age applicability during the 4–6-year-old stage.

Furthermore, the type of donation item (stickers vs. candies) did not produce a significant difference in children’s pro-environmental behavior (supporting H4). This indicates that for the children in this study, both stickers and candies were perceived as highly attractive personal resources, and forgoing either represented a real cost. This result enhances the validity of the experimental paradigm, suggesting that the measured pro-environmental donation behavior reflects a relatively stable altruistic tendency, not driven by preferences for specific items.

In summary, Experiment 1 demonstrated at the behavioral level that inducing empathy with nature can directly and effectively promote pro-environmental behavior in young children, and this effect shows cross-age stability during the middle-to-late preschool years. This finding not only provides new ecological validity evidence for empathy theory in developmental psychology but also clarifies the core value of affective priming in early environmental education. It establishes a solid behavioral foundation for Experiment 2, which uses eye-tracking technology to further investigate the immediate attentional processing mechanisms behind this affective influence.

## 3 Experiment 2: the attentional mechanism of empathy with nature on pro-environmental behavior in young children: an eye-tracking study

### 3.1 Research purpose and hypotheses

Experiment 1 provided robust behavioral evidence that inducing empathy with nature effectively promotes pro-environmental donation behavior in 4–6-year-olds, and demonstrated that this effect is consistent across age groups. However, behavioral data alone cannot reveal the underlying cognitive processes that translate an induced affective state (empathy) into a concrete behavioral choice (donation). Experiment 2 aimed to employ eye-tracking technology to reveal the attentional mechanism through which empathy with nature influences children’s pro-environmental behavior, by recording the real-time gaze patterns of 5-year-olds during a resource allocation decision-making task. Building directly on the foundation established in Experiment 1, this experiment specifically investigated whether inducing empathy with nature promotes more pro-environmental decisions by enhancing children’s attentional bias toward the pro-environmental option.

Based on the theory of prioritized processing of emotional information and related research on prosocial decision-making, the following hypotheses were proposed:

*H5*: (Behavioral Replication Hypothesis): In a computerized donation task, children in the induced-empathy condition would donate significantly more stickers than those in the control condition, replicating the core finding of Experiment 1.

*H6*: (Attentional Bias Hypothesis): During the presentation of the decision interface, compared to the control group, children in the induced-empathy condition would show a stronger attentional bias toward the area of interest (AOI) representing the “donate to the environmental organization” option. This would be reflected in a higher ratio of total fixation duration and a higher ratio of total fixation count on that AOI.

*H7*: (Attention-Behavior Link Hypothesis): Children’s ratio of fixation duration and ratio of fixation count on the donation AOI would both be positively correlated with their actual number of stickers donated.

### 3.2 Methodology

#### 3.2.1 Participants

Five-year-old children in good health were selected as participants. An a priori power analysis was conducted using G*Power 3.1 (Faul et al., 2007). For an independent-samples *t*-test, with α = 0.05, power (1–β) = 0.80, and an expected medium-to-large effect size (*d* = 0.80), the analysis indicated a required minimum of 21 participants per group. Accounting for potential data loss common in eye-tracking studies with young children, a total of 61 5-year-olds were successfully recruited (*Mag*e = 65.3 months, SD = 3.5; 30 boys, 31 girls). Participants were randomly assigned to either the control condition (*n* = 31) or the induced-empathy condition (*n* = 30). All participants had normal or corrected-to-normal vision, no color vision deficiency, and no history of neurodevelopmental disorders. Written informed consent was obtained from guardians, verbal assent was obtained from the children, and the study was approved by the institutional ethics review board.

#### 3.2.2 Experimental design

A single-factor (Condition: induced empathy with nature vs. control) between-subjects design was employed.

Independent variable: Empathy priming condition (between-subjects).

Dependent variables:

Behavioral measure: The number of stickers for which the child chose the “donate to the environmental organization” option.

Eye-tracking measures: During the presentation of the decision interface: (a) Ratio of total fixation duration on the donation AOI (time spent fixating on the donation AOI/total time spent fixating on the decision interface); (b) Ratio of total fixation count on the donation AOI (number of fixations landing on the donation AOI/total number of fixations on the decision interface).

#### 3.2.3 Experimental materials

Empathy priming materials: identical to those used in Experiment 1, comprising four pictures for the induced-empathy condition (suffering animals) and four for the control condition (animals in natural states).

Eye-tracking stimuli and behavioral task: the experimental procedure was programmed using Tobii software. A single trial consisted of: (1) a central fixation cross “+” presented for 500 ms; (2) a full-screen priming picture presented for 5 s; (3) the decision interface, presented until the child responded. The decision interface displayed the text “Your favorite stickers” at the top. Below it, two equally sized colored icon options were presented side-by-side: one icon represented “donate to the environmental organization” (pro-environmental option), and the other represented “keep for myself” (self-benefiting option). The left/right position of the options was counterbalanced across participants.

Eye-tracker: a Tobii Pro Fusion screen-based eye-tracker with a sampling rate of 250 Hz was used. This device is relatively tolerant to head movements, making it suitable for research with young children. Stimuli were presented on a 24-inch LCD monitor with a resolution of 1,920 × 1,080 pixels.

Areas of interest (AOIs): two key AOIs were defined on the decision interface, covering the “donate to the environmental organization” icon and the “keep for myself” icon, respectively, for subsequent eye-tracking data analysis.

#### 3.2.4 Procedure

The experiment was conducted in a quiet room within the kindergarten with constant lighting. The procedure was as follows:

Preparation and calibration: The experimenter guided the child to sit comfortably in front of the eye-tracker, with their chin on a chin rest and eyes approximately 60 cm from the screen. A 9-point calibration procedure was conducted, aided by cartoon animations to attract the child’s gaze. The session proceeded only when calibration achieved a “good” or “excellent” rating.

Practice phase: Practice trials using neutral pictures and decision options were administered. This ensured the child understood the procedure—either “look at the option, after which the experimenter will press a key” or “point directly to the choice” (the response mode was chosen based on the child’s capability)—and comprehended the meaning of the two options.

Main experiment: The trial sequence described above was followed. During the decision phase, the child viewed the interface freely. The experimenter recorded the choice via keyboard based on the child’s clear verbal instruction or pointing gesture.

Conclusion phase: Upon completion, children received a number of stickers corresponding to their in-task “keep for myself” choices as compensation, along with a small gift. The entire session lasted approximately 15 min.

**FIGURE 6:**
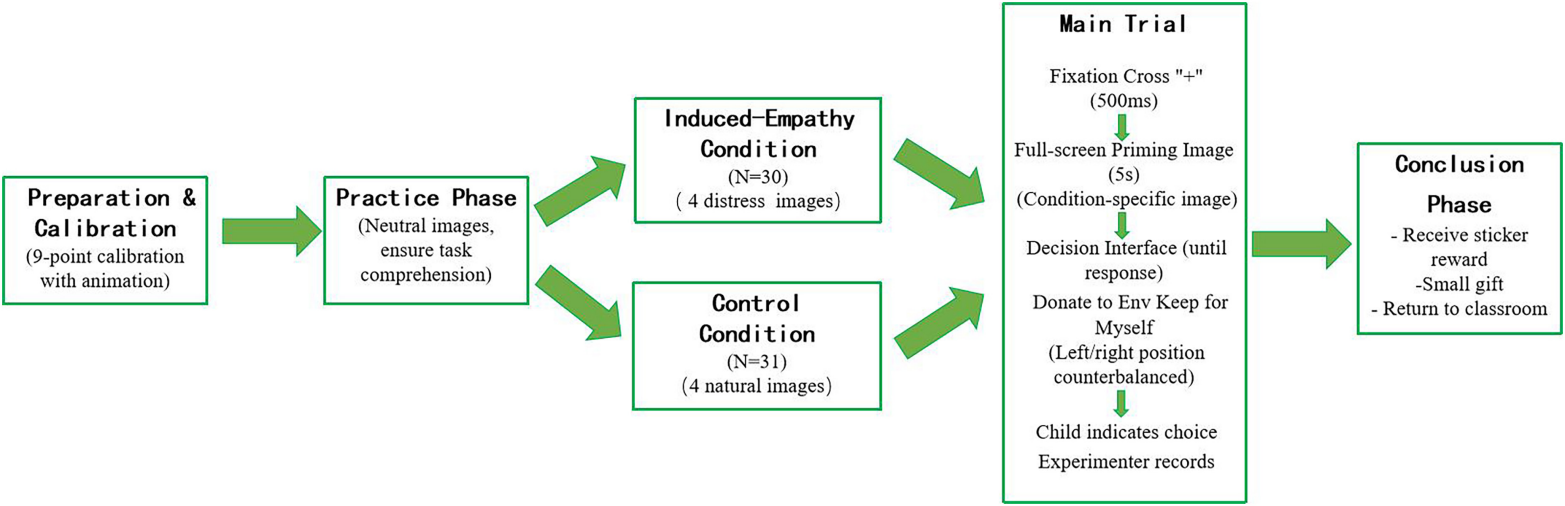
Flowchart of the experimental procedure for Experiment 2.

Figure 6 presents a flowchart illustrating the sequence of events in Experiment 2, including calibration, practice trials, and the main trial structure (fixation cross → priming picture → decision interface).

### 3.3 Results and analysis

#### 3.3.1 Behavioral results

To examine the effect of empathy induction on pro-environmental behavior in 5-year-olds, an independent-samples *t*-test was conducted with the number of stickers donated as the dependent variable. The results showed that children in the induced-empathy condition donated significantly more stickers (*M* = 2.67, *SD* = 1.27) than those in the control condition (*M* = 1.77, *SD* = 0.92), *t*(59) = 3.15, *p* = 0.003, Cohen’s *d* = 0.81, 95% CI (0.33, 1.46). Hypothesis H5 was supported, successfully replicating the core finding of Experiment 1 within the eye-tracking experimental context. This indicates a high degree of stability for the promoting effect of empathy with nature.

#### 3.3.2 Eye-tracking results

To investigate the effect of empathy induction on attention allocation during decision-making, independent-samples *t*-tests were conducted separately with the ratio of total fixation duration and the ratio of total fixation count on the donation AOI (environmental organization) as dependent variables.

Descriptive statistics showed that children in the induced-empathy condition had a significantly higher ratio of total fixation duration on the donation option (*M* = 0.47, *SD* = 0.13) compared to the control condition (*M* = 0.26, *SD* = 0.13), *t*(59) = 5.90, *p* < 0.001, Cohen’s *d* = 1.51, 95% CI (0.14, 0.28).

Similarly, the ratio of total fixation count on the donation option was also significantly higher for the induced-empathy group (*M* = 0.47, *SD* = 0.13) than for the control group (*M* = 0.26, *SD* = 0.13), *t*(59) = 5.90, *p* < 0.001, Cohen’s *d* = 1.51, 95% CI (0.14, 0.28). Descriptive statistics for the eye-tracking measures are presented in Table 3.

**TABLE 3:** Descriptive statistics for behavioral and eye-tracking measures by condition.

Group	n	Number of stickers to donate	Focus time ratio	Focal point ratio
No natural empathy group	31	1.77 ± 0.92	0.26 ± 0.13	0.26 ± 0.13
Natural empathy group	30	2.67 ± 1.27	0.47 ± 0.15	0.47 ± 0.15

In summary, Hypothesis H6 was fully supported: inducing empathy with nature significantly enhanced children’s visual attentional bias toward the pro-environmental option during decision-making.

#### 3.3.3 Correlation analysis between eye-tracking measures and behavioral results

**FIGURE 7:**
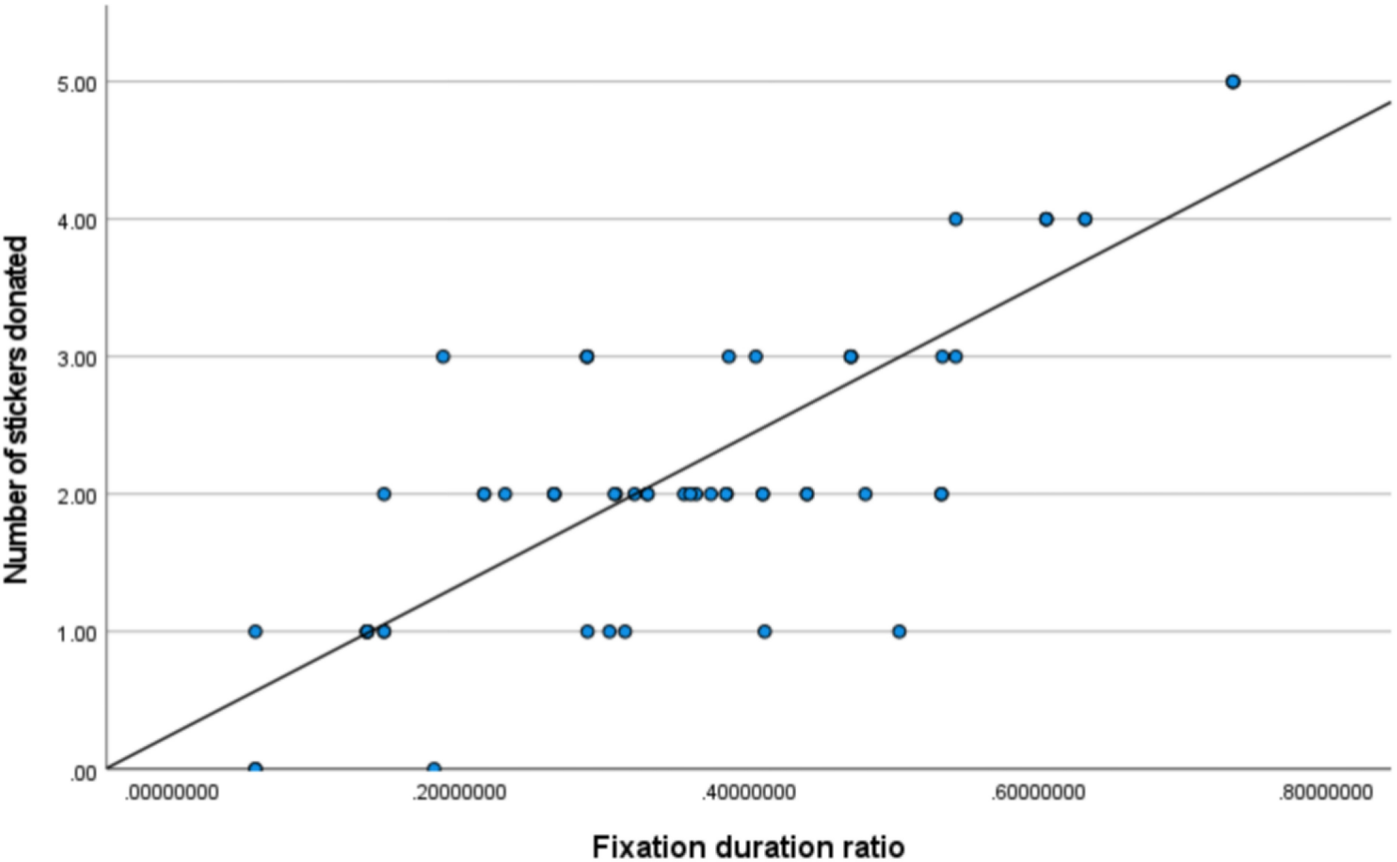
Correlation between fixation duration ratio and donation quantity.

**FIGURE 8:**
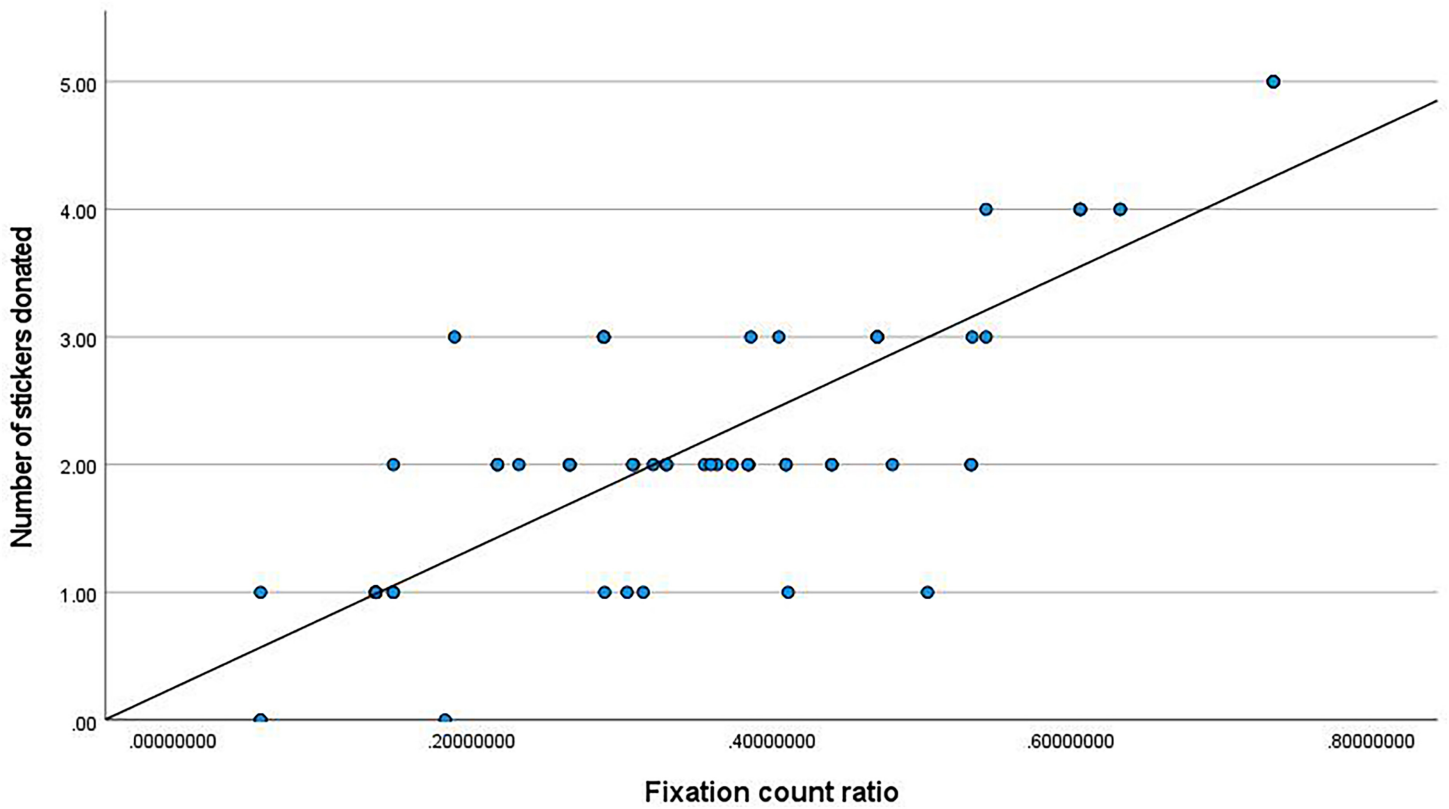
Correlation between fixation count ratio and donation quantity.

To examine the association between attentional bias and pro-environmental behavior, Pearson correlation analyses were conducted on the entire sample between the number of stickers donated and the two eye-tracking measures. The results revealed a strong positive correlation between the ratio of fixation duration on the donation option and the number of stickers donated, *r*(59) = 0.79, *p* < 0.001 (see Figure 7). Similarly, a strong positive correlation was found between the ratio of fixation count on the donation option and the number of stickers donated, *r*(59) = 0.81, *p* < 0.001 (see Figure 8).

These results provide strong support for Hypothesis H7, indicating that the degree of attentional engagement with the pro-environmental option during decision-making effectively predicts the ultimate behavioral choice, thereby offering direct evidence for an “attention-behavior” link.

### 3.4 Discussion

Experiment 2 aimed to employ eye-tracking technology to delve into the cognitive mechanisms through which empathy with nature influences young children’s pro-environmental behavior, specifically from the perspective of real-time attentional processing. The experimental results not only successfully replicated the promoting effect of empathy at the behavioral level but, for the first time in this age group, revealed a significant accompanying attentional bias behind this behavioral effect, along with a strong association between attention and behavior. This provides crucial process-level evidence for understanding the “empathy-pro-environmental behavior” pathway.

First, this experiment successfully replicated the stable promoting effect of empathy with nature on pro-environmental behavior within a standardized computerized task. Children in the induced-empathy condition donated significantly more stickers than those in the control condition. This finding once again strongly supports the applicability of the “empathy-altruism” hypothesis (Batson, 2014) to the domain of young children’s relationship with nature and robustly extends this conclusion to a more controlled experimental setting. It demonstrates that even a brief visual-emotional priming is sufficient to motivate preschoolers to sacrifice personal resources for environmental protection, highlighting the core role of emotional arousal in shaping early pro-environmental tendencies (Schultz, 2000).

Second, and central to this experiment, the eye-tracking data provided direct evidence for the hypothesis that “empathy with nature influences behavior by shaping attentional bias.” Compared to the control group, children in the induced-empathy condition exhibited a significantly stronger attentional bias toward the “donate to the environmental organization” option during decision-making, reflected in higher ratios of both total fixation duration and fixation count, with a very large effect size (Cohen’s *d* = 1.51). This result aligns with the view from the theory of prioritized processing of emotional information, which posits that motivationally or emotionally relevant stimuli can automatically capture and hold attentional resources (Öhman et al., 2001). In this study, the induction of empathy with nature may have tagged the “environmental donation” option as a more affectively or morally relevant goal, thereby guiding children to allocate more visual attentional resources to its evaluation during decision-making. This offers a clear cognitive mediating pathway for how empathy transforms from an internal affective state into a concrete behavioral tendency—namely, by modulating real-time attention allocation during the decision process.

Third, the strong correlations between the eye-tracking measures and behavioral outcomes further solidify the explanatory power of the attentional mechanism. Both the ratio of fixation duration and the ratio of fixation count on the donation option were highly positively correlated with the actual number of donations (*r*s > 0.79). This robust association indicates that the degree of an individual’s visual engagement with the pro-environmental option on the decision interface is a sensitive predictor of their ultimate behavioral choice. This not only corroborates the bridging role of attention in linking affective priming to behavioral output (Decety, 2021) but also suggests that in early childhood, attentional patterns during decision-making can already stably reflect the strength of prosocial motivation.

In summary, by combining behavioral and eye-tracking techniques, Experiment 2 paints a more detailed picture of the underlying mechanism: inducing empathy with nature not only directly enhances the outcome of children’s pro-environmental behavior but also, by guiding them to allocate more attentional resources toward the pro-environmental choice when faced with the “self-benefiting” versus “environment-benefiting” decision conflict, increases the likelihood of making that choice. This finding deepens the conclusion of Experiment 1 at the cognitive-process level, concretizing the affective drive into an observable attentional bias, thereby providing novel and direct evidence for understanding the generation mechanism of pro-environmental behavior in young children. Furthermore, the results offer an implication for early childhood environmental education practice: while fostering empathy with nature in children, guiding them to notice and recognize the value of pro-environmental behavioral options may, by shaping a positive attentional preference, more effectively promote the development of their pro-environmental habits.

## 4 General discussion

### 4.1 Optimistic attributional style significantly inhibits college students’ impulsive consumption

Through two experiments integrating behavioral measurement and eye-tracking technology, this study systematically examined the influence of empathy with nature on pro-environmental behavior in 4–6-year-old children and its underlying attentional mechanism. Experiment 1 established the basic behavioral phenomenon: inducing empathy with nature significantly promoted pro-environmental donation behavior across different age groups, with this effect showing cross-age stability between 4 and 6 years, while the baseline level of pro-environmental behavior increased with age. Experiment 2 then extended these findings by investigating the cognitive mechanism: focusing on 5-year-olds, it replicated the behavioral effect and, for the first time, revealed at a process level that empathy with nature promotes behavior by enhancing children’s visual attentional bias toward the pro-environmental option. Collectively, this research not only provides robust causal evidence that empathy with nature is a key affective driver of young children’s pro-environmental behavior but also deepens the understanding of this early developmental mechanism by uncovering the attentional mediating pathway.

First, this study offers compelling developmental psychological evidence for the “empathy-altruism” hypothesis (Batson, 2014) in the domain of children’s relationship with nature. Our findings demonstrate that even during the preschool years, when cognitive and moral reasoning abilities are still developing, children are capable of translating empathetic concern for suffering natural entities (the behavioral promotion effects in Experiments 1 and 2) into tangible resource-sacrificing actions. This result robustly extends conclusions previously based primarily on adults and adolescents (Berenguer, 2007; Schultz, 2000) to early childhood, confirming the foundational role of emotional resonance in shaping environmental tendencies early in life. The cross-age stability of this effect (indicated by the non-significant interaction) suggests that affect-based interventions hold potential efficacy across preschool age groups, providing a basis for designing environmental education programs targeting broad populations of young children.

Second, and constituting the core contribution of this study, the use of eye-tracking technology revealed the specific cognitive pathway through which empathy with nature influences behavior. Experiment 2 discovered that empathy induction not only altered behavioral outcomes but also changed the pattern of visual attention allocation at the moment of decision-making, manifested as significantly increased fixation duration and count on the pro-environmental option. This result is consistent with the theory of prioritized processing of emotional information (Öhman et al., 2001), indicating that the affective state aroused by empathy can tag the environmental option as a goal with higher motivational salience, thereby automatically attracting and sustaining more cognitive resources. More importantly, the strong correlations between gaze metrics and donation quantity (*r*s > 0.79) indicate that this attentional bias is not merely an epiphenomenon but serves as a key bridge connecting the internal affective state (empathy) with the external behavioral choice (donation) (Decety, 2021). This refines the relatively generalized notion of “affective drive” in prior research into an observable chain of “affective priming → attentional bias → behavioral choice” at the process level, providing new evidence for understanding the micro-mechanisms of prosocial decision-making in young children.

Third, this study illuminated the developmental characteristics of children’s pro-environmental behavior. Experiment 1 found that while the promoting effect of empathy was consistent across ages, the baseline level of pro-environmental behavior increased significantly with age, with a particularly notable rise between ages 4 and 5. This likely reflects the synergistic development of multiple socio-cognitive capacities. On one hand, children’s affective and cognitive empathy (perspective-taking) abilities develop throughout the preschool years (Decety and Svetlova, 2012), enabling a deeper understanding of environmental consequences. On the other hand, advancements in executive functions (e.g., impulse control, delay of gratification) (Blake et al., 2014) allow for more altruistic trade-offs between immediate personal rewards and future/other-oriented environmental benefits. Future research could further disentangle the specific contributions of these cognitive components.

The findings of this study carry clear implications for early childhood environmental education practice. First, educators should recognize the value of emotional arousal as an entry point. Approaches that elicit empathy, such as telling nature stories or watching ecological documentaries, can serve as effective starting points for stimulating children’s environmental willingness. Second, building upon evoked empathy, educators should consciously guide children’s attention to the “pro-environmental behavioral options” themselves. For instance, in role-playing or game design, highlighting the availability and positive outcomes of environmental choices may help shape a positive attentional preference, thereby channeling affective motivation smoothly into concrete actions.

## 5 Conclusion

This study, combining behavioral and eye-tracking experiments, demonstrates that empathy with nature stably promotes pro-environmental behavior in 4–6-year-olds, primarily by enhancing a visual attentional bias toward pro-environmental options. The promoting effect of empathy was consistent across ages, while the baseline level of pro-environmental behavior increased with age. These findings provide direct evidence for the affective drivers of early prosocial development and offer clear implications for empathy-based environmental education.

## Data Availability

The original contributions presented in this study are included in this article/supplementary material, further inquiries can be directed to the corresponding author.
